# High-speed all-optical neural networks empowered spatiotemporal mode multiplexing

**DOI:** 10.1038/s41377-025-02007-5

**Published:** 2025-09-25

**Authors:** Fu Feng, Xiaolong Li, Ziyang Zhang, Jiaan Gan, Xiaocong Yuan

**Affiliations:** 1 Research Center for Frontier Fundamental Studies, Zhejiang Lab, Hangzhou, China; 2 State Key Laboratory of Extreme Photonics and Instrumentation, College of Optical Science and Engineering, Zhejiang University, Hangzhou, China; 3 College of Electrical Engineering, Zhejiang University of Water Resources and Electric Power, Hangzhou, China; 4 Institute of Modern Optics, Nankai University, Tianjin, China

**Keywords:** Fibre optics and optical communications, Ultrafast photonics, Imaging and sensing

## Abstract

Orbital angular momentum (OAM) beams, characterized by a helical phase structure and phase singularity, have emerged as a powerful resource for high-capacity optical communications through mode-division multiplexing (MDM). Traditional OAM multiplexing systems operating solely in the spatial domain face significant challenges, including increased system complexity, inter-modal crosstalk, and limited scalability. Recent advances have explored hybrid multiplexing schemes combining OAM with wavelength or polarization degrees of freedom, demonstrating Pbit/s level transmission capacities. However, these systems predominantly rely on continuous-wave lasers and external modulators, which constrain their applicability in challenging environments, whereas pulsed lasers provide superior peak power, enhanced transmission robustness, and the potential for implementation of OAM lasers, which generally emit pulsed OAM beams. Here, we report an OAM-based spatiotemporal multiplexing (OAM-STM) technique that synergistically implements pulsed OAM beams with a diffractive deep neural network (D^2^NN) and optical fiber delay lines to project spatial mode information into the temporal domain. This approach leverages the full potential of pulsed laser sources by activating the underutilized time dimension, thereby overcoming the repetition-rate bottleneck and enhancing channel throughput. We experimentally demonstrate an OAM-based spatiotemporal demultiplexer achieving demultiplexing speed limited only by the bandwidth of the photodiode if OAM generation is fast enough. In the meantime, the architecture is intrinsically compatible with high-repetition-rate OAM sources, offering the entire system the scalability to GHz rates. This work establishes a foundational framework for high-speed, all-optical, and high-capacity OAM-STM systems, with promising implications for free-space optical communication, underwater communication links, and other complex environments.

## Abstract (web summary)

We have developed an OAM-based spatiotemporal multiplexing system using an all-optical neural network, demonstrating the tremendous potential of OAM-based multidimensional multiplexing methods for high-speed and large-capacity optical communications.

## Graphical abstract

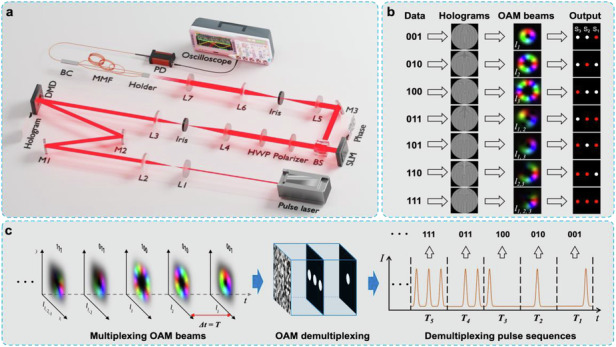

## Introduction

Light beams carrying orbital angular momentum (OAM), described by $\exp ({il}\theta )$^1^, where *θ* is the azimuthal angle and *l* represents the topological charge, are known as vortex beams or OAM beams. Their phase singularity and ring-shaped intensity profile impart unique optical properties, enabling applications in nanoparticle manipulation^2,3^, spatiotemporal light structuring^4–8^, quantum information^9,10^, and optical communications^11,12^. Moreover, in communication systems, OAM facilitates mode-division multiplexing (MDM), providing an additional degree of freedom beyond traditional multiplexing methods such as wavelength^13^, polarization^14^, time^15^, and space^16^. Owing to the theoretically infinite set of orthogonal OAM modes, this approach holds significant potential for further enhancing communication capacity and transmission speed.

Conventional OAM-multiplexed communication systems typically enhance the overall channel capacity by increasing the number of orthogonal OAM modes that can be simultaneously multiplexed and demultiplexed. Common demultiplexing techniques include interferometric methods^17–19^, phase plates^20–22^, Dammann gratings^22,23^, log-polar coordinate transformations^24,25^ and deep learning method^26–28^. These approaches operate solely in the spatial domain and face inherent limitations, including increased system complexity, growing crosstalk with the number of OAM modes, and restricted demultiplexing efficiency. As a result, relying solely on the OAM modal dimension poses fundamental challenges in scaling up to high-capacity optical communication systems. To overcome these limitations, recent studies have explored combining OAM multiplexing with other degrees of freedom to boost overall communication capacity. For instance, Yan et al. developed a millimeter-wave communication system that integrates OAM and polarization multiplexing, enabling simultaneous transmission of multiple OAM beams over the same frequency and propagation path, achieving a data rate of up to 32 Gbit/s^29^. Zou et al. proposed a mid-infrared free-space optical communication system that combines wavelength division multiplexing (WDM) with MDM, transmitting two distinct OAM modes across three adjacent wavelengths around 3.4 μm, resulting in an aggregate rate of 300 Gbit/s^30^. Pushing the boundaries further, researchers have demonstrated a multidimensional multiplexed fiber-optic communication system by integrating OAM-MDM with WDM, achieving a record-breaking total capacity exceeding Pbit/s for an OAM-based link^31,32^. These advances underscore the substantial potential of multidimensional multiplexing schemes that combine OAM mode with other physical degrees of freedom for enabling ultra-high-capacity optical communication networks. Notably, most of the reported implementations rely on continuous-wave (CW) laser sources combined with external modulators to generate the desired OAM states.

While such configurations are well-suited for standard applications, they present limitations in certain application scenarios where pulsed laser sources are preferred. Pulsed lasers offer distinct advantages, particularly in complex or long-distance environments, due to their exceptionally high peak power and enhanced robustness against environmental impairments such as atmospheric turbulence. These features make pulsed sources especially attractive for free-space optical communication^33,34^, underwater wireless optical communication^35,36^, and quantum communication^37,38^. Moreover, many state-of-the-art OAM generators^39–41^ inherently operate in pulsed mode and are capable of directly emitting OAM beams without the need for additional external modulators. This direct generation significantly reduces system complexity and facilitates on-chip integration, which is often difficult to achieve with CW-based schemes. A major challenge with pulsed-laser-based systems lies in the temporal structure of the emitted signal: the time interval between adjacent pulses is fixed by the laser repetition rates, which can lead to underutilization of the temporal channel. This constraint has limited the data throughput of pulsed OAM systems in such scenarios, despite their physical advantages.

To overcome the aforementioned limitations, we propose a novel OAM-based spatiotemporal multiplexing (OAM-STM) method that integrates a diffractive deep neural network (D^2^NN) with optical fiber delay lines to project the spatial distribution of OAM beams into the temporal domain for spatiotemporal multiplexing communication. By introducing time-domain into spatial optical neural networks, this approach not only leverages the intrinsic advantages of pulsed lasers but also activates the otherwise underutilized temporal degree of freedom, thereby enhancing overall data throughput. The experimental system successfully demonstrated an OAM-based spatiotemporal multiplexor of which the demultiplexing speed is theoretically only limited by the bandwidth of the photodiode. Although the entire system experimentally operates at kHz rates (limitation arises solely from the use of a relatively slow digital micromirror device), the underlying OAM-STM architecture is inherently compatible with high-speed OAM generators, making it readily scalable to GHz speeds. This highlights the strong potential of the proposed approach to drive OAM-STM communication into the ultrafast and high-capacity domain, paving the way for next-generation optical interconnects and real-time data transmission in complex environments.

## Results

### Principles of OAM-STM

**Fig. 1:**
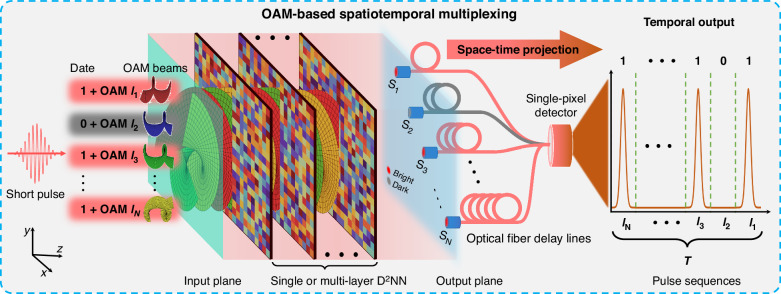
Conceptual illustration of OAM-STM. By encoding information into OAM channels using pulsed light, multiplexing OAM beams propagate in free space and through a trained single or multi-layer D^2^NN. Each OAM eigenmode is focused onto distinct spatial regions, in which the light is delayed and projected into the temporal domain using multiple optical fiber delay lines. A single-pixel detector detects the temporal signals of all the delayed light

Figure 1 illustrates the principle of the proposed OAM-STM method based on a high-speed all-optical neural network. A pulsed light source is employed as the information carrier, in which each N-bit data is encoded into an OAM state. Following the input plane, each OAM eigenmode propagates and intermixes before entering a single or multi-layer D^2^NN. Each diffractive layer within this optical network can be optimized using the error backpropagation algorithm (see Supplementary Material 1 for the principle of the D^2^NN). These optimized diffractive layers each OAM eigenmode in the mode-mixed OAM beams to be focused at distinct spatial regions on the network’s output plane. After that, each spatial region is connected with an optical fiber delay line, which couples and transmits the light focused onto that specific region. Each optical fiber delay line has a different length, introducing distinct delays to the incoming light. Finally, the signals from all the optical fiber delay lines are combined and then detected using a single-pixel detector. Due to the light signals entering the optical fiber delay lines from each spatial region being output in the form of pulse signals, the final output of the system is a sequence of pulses obtained by summing the outputs from each optical fiber delay line. During this process, the spatial information of OAM beams is projected into the temporal domain and output as the pulse sequences. Due to the different time delays applied to the light focused into each spatial region, each OAM eigenmode is projected into different time intervals within a pulse period of the laser. Therefore, the input information carried in OAM beams can be inferred directly from the distribution of the temporal pulse sequences. In this way, the speed of OAM-STM is expected to increase to the bandwidth limit of single-pixel detectors.

### Experimental setup of OAM-STM system

**Fig. 2:**
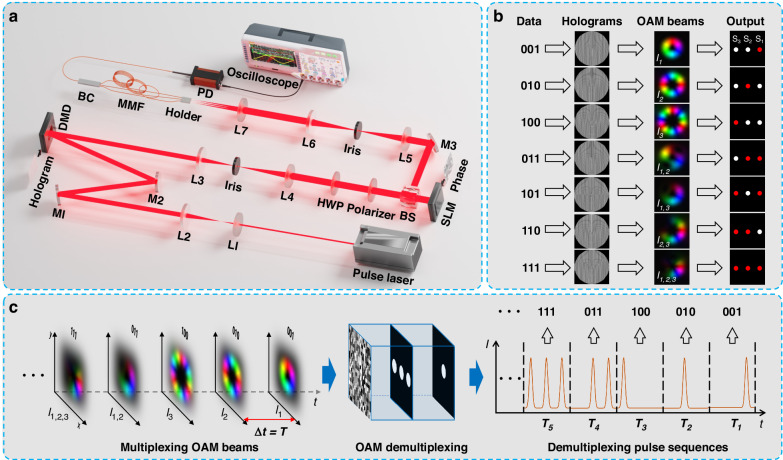
Schematic diagram of the OAM-STM system based on D^2^NN. **a** Experimental setup: L1~L7: plano-convex lens, M1~M3: mirrors, HWP half-wave plate, BS beam splitter, MMF multimode fiber, BC 3 × 1 beam combiner, PD InGaAs photodetector. **b** Spatial encoding/decoding rules for 3-bit data. **c** Operation diagram of the OAM-STM system

To evaluate the feasibility of the proposed OAM-STM method, a proof-of-concept system was constructed, as illustrated in Fig. 2a (the detailed experimental setup is described in the Materials and Methods). It should be noted that a single-layer D^2^NN was used in the experimental demonstration. Due to the limited modulation capacity of a single-layer network, and to ensure fully accurate decoding, the experiment only demonstrated the encoding and decoding of 3-bit data corresponding to the multiplexing and demultiplexing of three OAM modes. In the demonstration, a mode-locked ultrafast pulsed laser (EKSPLA, FPS200) operated at 1064 nm with a pulse duration of 10 ps was utilized as the light source. The laser beam was collimated and expanded by a 4 f system before being incident onto the patterns displayed on the digital micromirror device (DMD). It is worth noting that the patterns displayed on the DMD are computer-generated holograms (CGHs) based on the principle of Lee hologram^42,43^ (the detailed calculated process of binary holograms is described in the Materials and methods). In this way, seven 3-bit data groups ranging from “001” to “111” are encoded into OAM beams with the topological charge *l*∈[1,3] for information multiplexing. The encoding process is illustrated in Fig. 2b, following the sequence “Data”-“Holograms”-“OAM beams”, where the data of “001”, “010” and “100” were encoded into OAM eigenmode *l*_1_, *l*_2_ and *l*_3_, respectively, “011”, “101”, “110” and “111” were encoded into superposition state of OAM mode *l*_1,2_, *l*_1,3_, *l*_2,3_ and *l*_1,2,3_ respectively.

The OAM beams generated by these holograms are filtered through a 4 f system, with the +1 order diffraction light directed onto a phase-only spatial light modulator (SLM). This SLM is used to construct the single-layer D^2^NN. The phase distribution loaded on the SLM is trained on computers using the error-backpropagation algorithm (the training process for the D^2^NN is available in Supplementary Material 2). The designated target light field intensity distribution is illustrated in the column “Output” of Fig. 2b, which features three horizontally arranged “activation regions” (S_1_, S_2_, and S_3_) on the output plane. Each “activation region” corresponds to an OAM eigenmode, arranging S_1_, S_2_, and S_3_ from right to left, corresponding to OAM *l*_1_, *l*_2_, and *l*_3_, respectively. When the OAM beam passes through the trained diffractive layer, the energy of each eigenmode mixed in the OAM beam is focused into its corresponding “activation region”, which is marked with red color.

At the detection end of the system, three multimode fibers (MMFs) with varying lengths are arranged, with the ends of the fibers coinciding with the output plane of the D^2^NN. These three MMFs constitute a set of optical fiber delay lines, each aligned with an “activation region”, allowing the light from each “activation region” to be separately coupled into a fiber for time delay operations. The output light signals from the optical fiber delay lines are combined and incident into an MMF via a 3 × 1 beam combiner (the coupling efficiency of each channel is listed in Supplementary Material 3), and detected by a high-speed photodetector (InGaAs PD, wavelength range: 800–1700 nm, bandwidth in −3dB: 5 GHz). The temporal pulse sequences are subsequently recorded by an oscilloscope (bandwidth: 32 GHz, sample rate: 80 GSa/s). These optical fiber delay lines are standard step-index MMFs featuring a core/cladding diameter of 200/220 μm, a numerical aperture (N.A.) of 0.22, and lengths of 2 m, 4 m, and 6 m, corresponding to the “activation regions” S_3_, S_2_, and S_1_, respectively. The single MMF connected to the PD has a core/cladding diameter of 200/230 μm, with an N.A. of 0.46, and a length of 0.2 m. The optical fiber delay lines and PD form a “spatiotemporal projector” (STP), which is capable of projecting spatial light fields into the temporal dimension and performing time-delay operations for different spatial distributions. It should be noted that the number of optical fiber delay lines in the STP can be adjusted based on the number of bits data, and the differential lengths between the fiber delay lines can also be modulated to precisely control the time delay for each channel.

Ultimately, the experimental setup for the OAM-STM system is capable of performing the functions depicted in Fig. 2c. When the input light field contains information pertaining solely to a single OAM eigenmode, it will be projected to its respective position in the temporal domain. Conversely, when the input light field comprises multiple mixed OAM modes, each eigenmode is separated and projected to its corresponding position in the temporal domain, thus forming a multi-pulse sequence with a specific distribution in a period. In this way, the input information can be directly decoded from the distribution of the output temporal pulse sequence.

### Performance of OAM-STM system

**Fig. 3:**
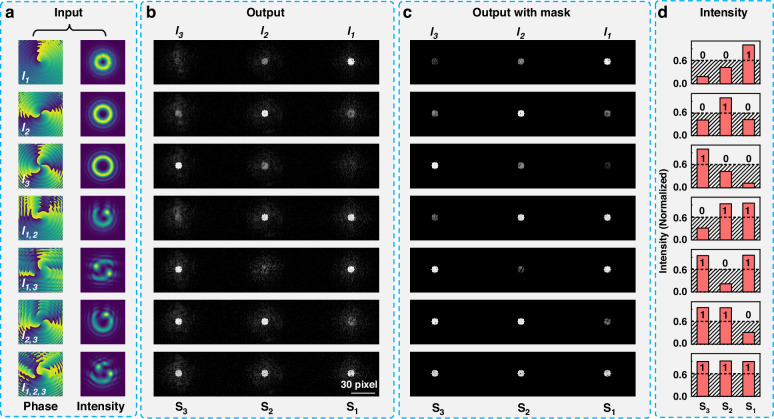
Simulation results of OAM demultiplexing. **a** Training dataset, including phase and intensity from the complex matrix calculated by the binary holograms, **b** Output intensity distribution of the D^2^NN, **c** Output intensity distribution with mask, whose sizes were designed to match three “activation regions”, **d** Normalized intensity values through summing the light intensity in each “activation region”, the intensity threshold was set at 0.6 to achieve 100% accuracy

Simulation and experimental investigations have been conducted to evaluate the performance of the OAM-STM system. Figure 3 presents the numerical simulation results of the D^2^NN. Figure 3a displays the dataset used for training the diffractive layers in the D^2^NN, comprising the phase and amplitude distributions of seven OAM beams carrying 3-bit data. The training dataset, derived from CGHs followed by preprocessing (including beam expansion and diffraction propagation), can be directly fed into the diffractive network for training. Figure 3b shows the output results obtained from training based on spatial encoding rules (see Supplementary Material 2 for the optimized phase distribution and evolution curve of the loss function). It can be observed that for the OAM beams with single eigenmode (*l*_1_, *l*_2_ and *l*_3_), despite light intensity is detected in all three “activation regions”, the light intensity corresponding to each input OAM eigenmode is significantly higher in its designed “activation region” compared to the other regions, indicating that the majority of the energy of each OAM eigenmode is focused into its corresponding “activation region”. For OAM beams with mixed modes (*l*_1,2_, *l*_1,3_, *l*_2,3_, and *l*_1,2,3_), each OAM eigenmode is spatially separated and focused into the corresponding “activation region”, displaying higher light intensities in multiple corresponding “activation region” correlating with the input. It is important to note that in subsequent experiments, the light from each “activation region” needs to be coupled into the fiber optic delay line. Therefore, it is necessary to extract the light intensity from each “activation region” in order to compare it with the signal strength obtained experimentally. The core size of the fiber delay lines is limited, and thus, each fiber’s input face acts like a circular mask that filters out light outside the core. The light intensity detected by the PD depends on the light intensity coupled into the core. Thus, to compare with the light intensities detected by the PD in the experiment, it is necessary to eliminate stray light outside the “activation regions” during the process of extracting light intensity from simulated results. Here, using the three designed “activation regions” shown in the “Output” column in Fig. 2b as masks to filter out stray light, the results are shown in Fig. 3c. By comparing the light intensity before and after applying the mask filter, it was found that the total intensity within the three “activation regions” accounts for approximately 91 ~ 94% of the total detected intensity across the entire output plane. This indicates that the diffractive network focuses the vast majority of energy into the three “activation regions”. Subsequently, the total light intensity within each “activation region” after masking is calculated, and a normalized intensity histogram for each type of OAM beam across the three “activation regions” is obtained, as shown in Fig. 3d. Setting an intensity threshold of 0.6, intensity values below this threshold are encoded as 0, and these above as 1, resulting in a spatial decoding of each OAM beam consistent with the input 3-bit data.

**Fig. 4:**
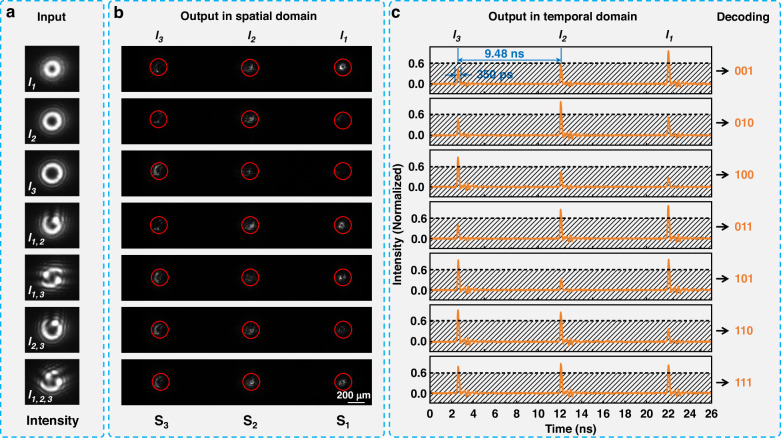
Experimental results of OAM demultiplexing. **a** Input intensity distribution detected by a CCD camera on the front face of the SLM by switching the binary holograms displayed on the DMD. **b** Output intensity distribution detected by a CCD camera on the front face of the optical fiber delay lines by switching the binary holograms displayed on the DMD. **c** Normalized waveforms recorded by an oscilloscope by switching the binary holograms displayed on the DMD, the intensity threshold was set at 0.6 to obtain the decoding results

Based on the simulations, the trained single-layer phase distribution was loaded onto the SLM for the experiment. The DMD was set to a constant display state, and the output results and their variations were observed by manually switching the binary holograms. The experimental results were depicted in Fig. 4, where Fig. 4a illustrates the light field intensity distribution of seven types of OAM beams detected in the front surface of the SLM, showing no significant differences compared to the intensity distributions simulated in Fig. 3a. Figure 4b presents the light field intensity distribution detected on the front face of the optical fiber delay lines (posterior focal plane of L7), the “activation regions” were marked with red dashed circles with a diameter of 200 μm, matching the core size of the optical fiber delay lines used in this experiment. By comparing the light intensity within the three “activation regions” to the total intensity detected by CCD, it was found that 82~86% of the light intensity is localized within the three “activation regions”, which demonstrates that the SLM is capable of focusing 82 ~ 86% of the energy into the cores of the optical fiber delay lines. Moreover, it is observed that the majority of energy from OAM beams with the single OAM eigenmode (*l*_1_, *l*_2_ and *l*_3_) is focused into the corresponding “activation region”, and the majority of energy from each OAM eigenmode in the mode-mixed beams (*l*_1,2_, *l*_1,3_, *l*_2,3_ and *l*_1,2,3_) is separated and focused into each corresponding “activation region”. Figure 4c shows the normalized time-domain waveforms recorded by an oscilloscope, where each peak from left to right corresponds to the light signals coupled from “activation regions” S_3_, S_2_, and S_1_, respectively, associated with the OAM eigenmodes *l*_3_, *l*_2_, and *l*_1_. It is evident that within each group of pulse sequences in the time-domain waveform, there are significant differences in the peak values of the three peaks, with beams containing OAM eigenmodes exhibiting higher peak values at corresponding waveform positions. When the intensity threshold is set to 0.6, values below this intensity threshold are encoded as 0, and those above are encoded as 1, the decoding information of 3-bit data was obtained from the temporal pulse sequences. The temporal decoding information is consistent with the input information in the spatial domain. The experimental results demonstrate that the constructed OAM-STM system is capable of performing OAM demultiplexing operations on spatially input OAM beams in the time domain.

Additionally, a fixed time delay of 9.48 ns between adjacent peaks was observed in Fig. 4c, with each peak having a width of 350 ps. The time delay between the peaks is determined by the length difference between the optical fiber delay lines (2 m in this experiment), while the width of the peaks is primarily governed by the convolution of the laser pulse duration and the detector’s response. The response time of the PD used in the experiment is approximately 350 ps, including both the rise and fall times, significantly longer than the 10 ps width of the laser pulse, thus the shape of the peaks resembles the response curve of the PD (the oscillation at the end of the falling edge of each peak originates from the “ringing effect” of the PD.). Therefore, each detection period is the sum of the widths of the three peaks and the time delay between them, totaling 9.53 ns. The time delay between the peaks can be further adjusted to zero by reducing the length difference between the optical fiber delay lines, potentially shortening the detection period.

### Performance of high-speed detection

**Fig. 5:**
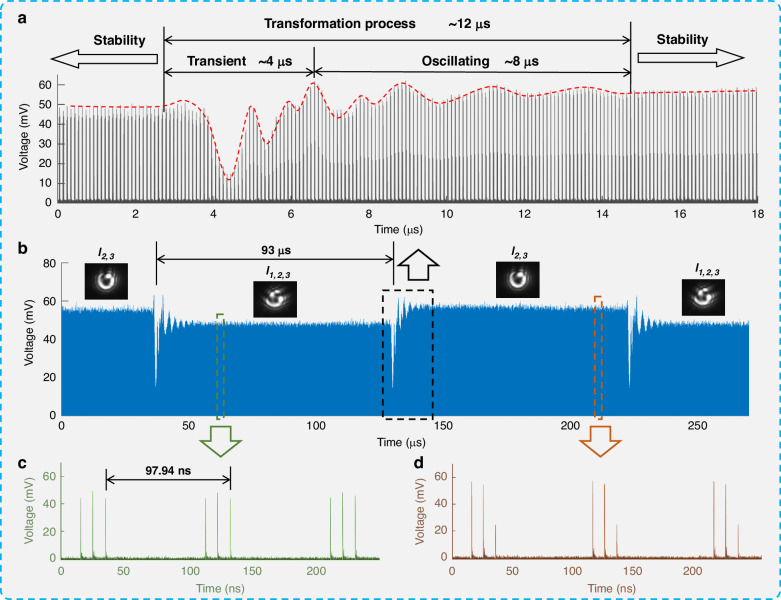
Experimental results of high-speed detection. **a** Waveforms resulting from the switching process of DMD displaying binary holograms corresponding to OAM modes with 3-bit data form “*l*_1,2,3_” to “*l*_2,3_”. **b** Waveforms recorded with the DMD periodically displaying two binary holograms corresponding to OAM modes “*l*_1,2,3_” and “*l*_2,3_”. **c** and **d** present the pulse sequences of OAM modes “*l*_1,2,3_” and “*l*_2,3_” in the temporal domain

To evaluate the performance of the OAM-STM system in high-speed detection, the laser pulse repetition frequency was set to 10.21 MHz to generate ultrafast OAM beams. However, the maximum switching rate of the DMD is limited to 10752 Hz, significantly lower than the pulse repetition rate of the laser. This discrepancy causes the production of a large number of OAM beams with the same mode within a single switching period of the DMD, preventing the realization of ultra-high-speed OAM demultiplexing. The switching from one image to another takes approximately 12 µs for the DMD, during which the mirrors undergo rapid flipping and brief oscillations. Therefore, the system was used to monitor this switching process to reveal the complex dynamics of the mirrors during this brief interval. The DMD operated at its highest switching rate, periodically displaying binary holograms corresponding to OAM modes “*l*_1,2,3_” and “*l*_2,3_”. The oscilloscope’s time range was set to 270 µs, enabling it to capture the entire sequence of high-speed events in a single recording. Figure 5b shows a frame of the time-domain waveform recorded by the oscilloscope. Since the DMD’s switching rate is three orders of magnitude lower than the pulse repetition rate of the laser, thousands of identical pulse sequences were captured within a single display period of 93 µs on the DMD. Each transformation behavior in the waveform indicates a switching process of the DMD. To facilitate analysis, waveform segments corresponding to the OAM modes “*l*_1,2,3_” and “*l*_2,3_” were extracted, along with the stepped waveforms resulting from the switching of binary holograms corresponding to OAM modes from “*l*_1,2,3_” to “*l*_2,3_”. Figure 5c, d (zoomed from the green and yellow rectangles marked in Fig. 5b, respectively) show the time-domain waveforms of OAM modes “*l*_1,2,3_” and “*l*_2,3_”, respectively. It can be observed that each group of pulse sequences has the same peak distribution as the corresponding pulse sequences in Fig. 4c, with each pulse sequence occurring at a period of 97.94 ns, corresponding to the repetition frequency of the laser pulses.

Figure 5a showcases the waveforms of the transformation behavior within 18 µs (zoomed from the black rectangle marked in Fig. 5b) corresponding to the switching process of the DMD. When plotting the upper envelope of the waveform, the overall trend of waveform changes becomes evident. It can be observed that, compared to the stable waveforms on both sides, the waveforms in the middle region undergo dramatic changes, which persist for 12 µs. This transformation process is consistent with the switching process of the DMD (see Supplementary Material 4 for the DMD switching process). This process can be divided into two stages. The first stage, termed the “transient” stage, lasts about 4 µs, during which the overall waveform exhibits a trend of first decreasing and then increasing, corresponding to the instantaneous switching of the DMD from one binary hologram to another. The second stage, termed the “oscillating” stage, lasts about 8 µs, during which the overall waveform undergoes a process from intense fluctuations to stabilization, caused by slight mechanical oscillations of the micromirrors after the DMD flipped. These results indicate that the speed of this system can fully characterize the complex switching dynamics of the DMD, but they also reveal that the switching rate of the DMD limits the overall operating speed of the system, which is far from reaching the system’s limit.

## Discussion

This work presents a spatiotemporal multiplexing paradigm that fundamentally rethinks how pulsed OAM modes can be exploited for high-speed optical communications. By introducing time-domain encoding into a spatially structured diffractive neural network and leveraging pulsed laser sources, this approach enables the simultaneous utilization of spatial and temporal degrees of freedom in an all-optical domain. The integration of MDM and time-domain multiplexing significantly enhances the utilization efficiency of a single temporal channel, thereby substantially increasing the total communication capacity. To experimentally validate the feasibility of the proposed OAM-STM scheme, we designed and implemented an experimental demonstration of the OAM-STM system. The diffractive layer parameters of the D^2^NN were optimized through numerical training, and an STP module was constructed using optical fiber delay lines and a single-pixel detector. This setup successfully projected OAM-encoded 3-bit data into temporal pulse sequences and performed OAM demultiplexing entirely in the optical domain. The system achieved a detection rate limited by the DMD switching rate (10,752 Hz) and is theoretically limited only by the bandwidth of the photodiode (~GHz).

At the moment, the system’s communication rate is primarily constrained by the low switching rate of the DMD and the limited repetition rate of the pulsed laser. These bottlenecks can be overcome by employing high-repetition-rate pulsed OAM generators or pulsed lasers, enabling OAM multiplexing at a GHz rate. Additionally, the communication capacity can be further enhanced by increasing the number of diffractive layers or the number of neurons in the D^2^NN, thereby allowing for higher-bit-rate data communication (the multi-layer simulation results are shown in Supplementary Material 5). The time delay difference in the current system can be further reduced to a level comparable to the output pulse width (~350 ps in our system), which requires only centimeter-scale in length of delay lines, it is possible to realize on-chip integration of the delay lines with high-refractive-index spiral waveguides or resonator-based optical delay lines^44,45^, offering compatibility with compact photonic circuits and potential integration into 5G all-optical networks^46–49^. Furthermore, the proposed OAM-STM architecture offers a versatile and scalable foundation for future high-capacity optical information systems, which unlocks new opportunities for surpassing the capacity limits of conventional multiplexing schemes.

## Materials and methods

### Experimental setup

In the experiment, the laser source used in the experiment was a pulse laser operated at a repetition rate of 10.21 MHz. The intensity distribution of the laser beam was a Gaussian profile, which was expanded and collimated using two lenses (L1 and L2). The expanded beam is reflected by two mirrors (M1 and M2) and then irradiates the DMD (Vialux V-650L, 10752 Hz switching rate with 1-bit input, 1280 × 800 resolution with 10.8 μm pixel size) at an incident angle of 12°. The DMD was comprised of a 2D array of micromirrors, each capable of toggling between on/off states. These states were determined by the orientation of each micromirror, which was in turn controlled through integrated circuits. Additionally, the independent control of each micromirror enables the arbitrary modulation of the spatial intensity distribution of the incident light field, leveraging the DMD’s capabilities for precise light manipulation. The DMD displays binary holograms (800 × 800 pixels) calculated by the Lee holography method. Specifically, due to the formation of multiple diffraction orders of OAM beams in space, a 4 f system (L3 and L4) and an iris were used to filter out diffraction orders other than the +1 order in the propagation path and to expand the beams again. In this way, OAM beams required in this experiment can be generated, and then vertically incident onto the SLM (Meadowlark Optics E19 × 12, 1920 × 1080 resolution with 8 μm pixel size) after passing through a half-wave plate and a polarizer. The functions of the half-wave plate and the polarizer were to adjust the polarization direction of the light field and increase the degree of polarization of the light field, respectively, so that the polarization direction of the incident light was consistent with the modulated direction of the SLM. The distance between the DMD and SLM is 0.7 m after subtracting the length of the 4 f system. It should be noted that in order to avoid the interference of the zero-order light field and the target light field of SLM, the two by adding a blazed grating to the loaded phase distribution diagram were spatially separated. After SLM modulation, a dichroic prism and a mirror (M3) reflect the outgoing light, and another 4 f system (L5 and L6) and an iris were used to filter out the diffracted light. After that, the light was focused by a lens into the input ends of the optical fiber delay lines. It should be emphasized that the input ends were fixed at the focal plane by a customized holder to realize the Fourier transform. The output light from the single MMF was detected by PD (Thorlabs, DET08CFC/M), and an oscilloscope (Keysight, DSAV164A) was used to record temporal light signals.

### Lee Holography method

Let $b\left(i,j\right)\in \left\{\mathrm{0,1}\right\},\left(1\le i\le m,1\le j\le n\right)$ be the pixels on the DMD, where 0 and 1refer to the “off” and “on” states of the micromirrors, respectively, and m and n refer to the number of rows and columns, respectively. According to Lee holography, the hologram on the DMD can be generated according to the following equation:

1 $$b\left(i,j\right)=\left\{\begin{array}{c}1,-\frac{{\sin }^{-1}A\left(x,y\right)}{2\pi }\le \frac{R\left(x,y\right)}{T}+\frac{\varphi \left(x,y\right)}{2\pi }+k\le \frac{{\sin }^{-1}A\left(x,y\right)}{2\pi }\\ 0,{\rm{ot}}{\rm{herwise}}\end{array}\right.$$

Where, $A\left(x,y\right)$ and $\varphi \left(x,y\right)$ are the amplitude and phase of the target light field, respectively, and x and y are the coordinates in the Cartesian coordinate system. $R\left(x,y\right)$ is a tilted phase to spatially separate different diffraction orders, *T* is the grating period, and *k* is an integer. Multiplexed OAM beams with 3-bit binary data need to be generated, the phase term of each OAM beam can be expressed in:

2 $${\varphi }_{h}\left(x,y\right)={B}_{h}[\exp \left(i{l}_{1}\theta \right)\exp \left(i{l}_{2}\theta \right)\exp \left(i{l}_{3}\theta \right)]\,h=\mathrm{1,2},\cdots ,7$$

Where, *B*_*h*_ is an 3 × 3 diagonal matrix $${\left[\begin{array}{ccc}a & 0 & 0\\ 0 & b & 0\\ 0 & 0 & c\end{array}\right]}_{h}$$, its eigenvalue [*a b c*]_h_ depends on the 3-bit binary data.

### Implementation of Loss Function

The intensity distribution of the output plane of the D^2^NN is set as shown in the “Output” of Fig. 2b. The output plane is divided into activation regions, where the value inside each region is set to 1, and the value outside is set to 0. Each activation region corresponds to an OAM intrinsic mode. To focus the OAM intrinsic modes contained in the input information onto their respective activation regions, during training, the actual output intensity distribution is compared with the predefined intensity distribution. The loss function is then defined based on the Pearson correlation coefficient *r*:

3 $${Loss}=1-r=1-\frac{{\sum }_{i=1}^{n}({A}_{i}-\widetilde{A})({B}_{i}-\widetilde{B})}{\sqrt{{\sum }_{i=1}^{n}{\left({A}_{i}-\widetilde{A}\right)}^{2}}\cdot \sqrt{{\sum }_{i=1}^{n}{\left({B}_{i}-\widetilde{B}\right)}^{2}}}$$

where *A*_*i*_ and *B*_*i*_ are the intensity distribution values for the output and target intensity distribution, respectively, and $\widetilde{A}$ and $\widetilde{B}$ represent the mean values of *A*_*i*_ and *B*_*i*_. This function ensures that the output intensity distribution closely matches the desired distribution for each OAM mode.

## Supplementary information

Supplementary Material

## Data Availability

All the data and methods needed to evaluate the conclusions of this work are presented in the main text and Supplementary Information. Additional data can be requested from the corresponding author.

## Code availability

The codes of the deep learning models reported in this work used standard libraries and scripts that are publicly available in TensorFlow.
