# Supplementary material for: High-speed all-optical neural networks empowered spatiotemporal mode multiplexing
Source: Light Sci Appl. 2025 Sep 25;14:342. doi: 10.1038/s41377-025-02007-5 (PMC12462434; doi:10.1038/s41377-025-02007-5)
Supplement: Supplementary file 1 — Supplementary Material [file 41377_2025_2007_MOESM1_ESM.docx]

**Supplementary Information for High-speed all-optical neural networks empowered** **spatiotemporal mode multiplexing**

Fu Feng^1, 2, *^, Xiaolong Li^1, 3^, Ziyang Zhang^1^, Jiaan Gan^4^, and Xiaocong Yuan^1, *^

^1^Research Center for Frontier Fundamental Studies, Zhejiang Lab, Hangzhou 311100, China

^2^State Key Laboratory of Extreme Photonics and Instrumentation, College of Optical Science and Engineering, Zhejiang University, Hangzhou 310027, China

^3^College of Electrical Engineering, Zhejiang University of Water Resources and Electric Power, Hangzhou 310018, China

^4^Institute of Modern Optics, Nankai University, Tianjin 300350, China

^*^Correspondence: Fu Feng ([fufeng@zhejianglab.org](mailto:fufeng@zhejianglab.org)), Xiaocong Yuan ([xcyuan@zhejianglab.org](mailto:xcyuan@zhejianglab.org))

These authors contributed equally: Fu Feng, Xiaolong Li and Ziyang Zhang

**Supplementary material 1: The principle of Diffractive Deep Neural Network**

In a Diffractive Deep Neural Network (D^2^NN), each point on a diffraction layer is treated as a secondary light source, influenced by the incident light wave and modulated by a complex-valued transmission or reflection coefficient. According to the Huygens-Fresnel principle, the position of each point and its connections to neurons in adjacent layers define the overall behavior of the network. These "neurons" interact with each other through diffraction and interference, forming a multi-layer optical neural network, as illustrated in Figure S1.


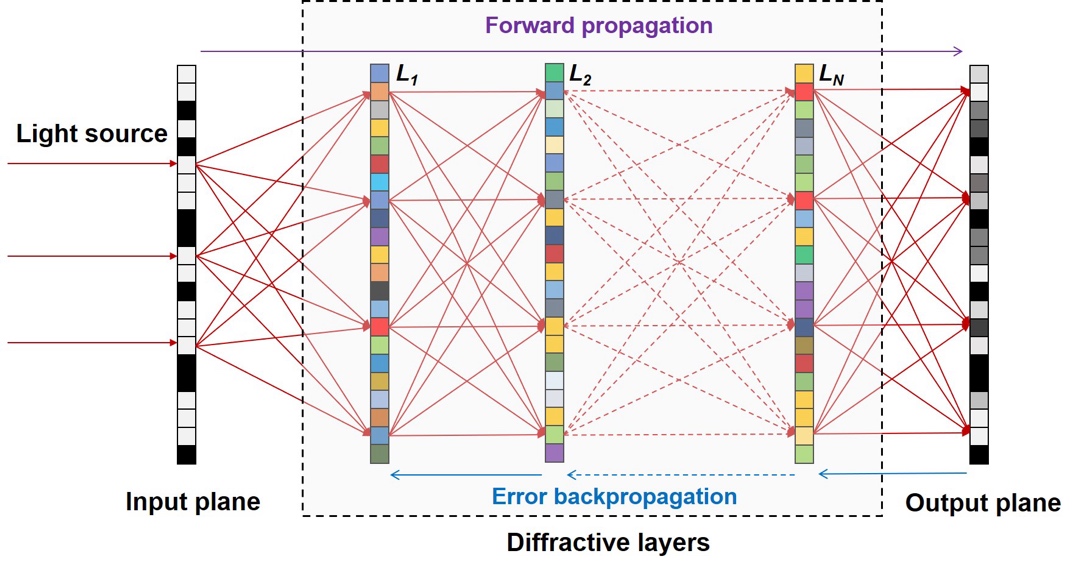


Figure S1. Schematic diagram of the principle of D^2^NN.

The light propagation between different diffraction layers in D^2^NN is described by the Rayleigh-Sommerfeld diffraction theory. At the  $l$-th diffraction layer, the  $i$-th neuron is located at $(x_{i},y_{i},z_{i})$, and each neuron's contribution to the diffraction is determined by the incident wave's propagation. The diffraction effect of each neuron can be expressed as:

$w_{i}^{l}\left( x,y,z \right)=\frac{z-z_{i}}{r^{2}}(\frac{1}{2\pi r}+\frac{i}{j\lambda})exp(\frac{j2\pi r}{\lambda})$ (1)

$$r=\sqrt{{(x-x_{i})}^{2}+{(y-y_{i})}^{2}+{(z-z_{i})}^{2}}$$

Here,  $r$ represents the distance between the neuron and the wave source, $\lambda$ is the wavelength of the incident wave, and $j$ is the imaginary unit. The light field at the$i$-th neuron modulated by the $l$-th layer can then be expressed as:

$u_{i}^{l}\left( x,y,z \right)=w_{i}^{l}\left( x,y,z \right)\cdot t_{i}^{l}(x_{i},y_{i},z_{i})\cdot\sum_{k\in M} u_{i}^{l-1}(x_{i},y_{i},z_{i})$ (2)

In this equation, $\sum_{k\in M} u_{i}^{l-1}(x_{i},y_{i},z_{i})$ represents the incident wave from the $(l-1)$-th layer, with $M$ being the set of all neurons in the previous layer. The complex-valued transmission or reflection coefficient at the $i$-th neuron is given by:

$t_{i}^{l}\left( x_{i},y_{i},z_{i} \right)=a_{i}^{l}\left( x_{i},y_{i},z_{i} \right)exp({j\phi}_{i}^{l}\left( x_{i},y_{i},z_{i} \right))$ (3)

where $a_{i}^{l}\left( x_{i},y_{i},z_{i} \right)$ is the amplitude and $\phi_{i}^{l}\left( x_{i},y_{i},z_{i} \right)$ is the phase of the coefficient. In the phase-only D^2^NN architecture, $a_{i}^{l}\left( x_{i},y_{i},z_{i} \right)$ is constant, while $t_{i}^{l}\left( x_{i},y_{i},z_{i} \right)$ can be adjusted by modifying the properties of the diffraction layer. This adjustment represents a learnable network parameter. During training, these parameters are iteratively optimized using gradient descent or error backpropagation algorithms.

**Supplementary material 2: Training process of D^2^NN**

The D^2^NN was trained using Python 3.6.5 and TensorFlow 2.4.0 on a high-performance server equipped with an NVIDIA GeForce RTX 3090 GPU and an Intel(R) Xeon(R) Silver 4210R CPU at 2.40 GHz, complemented by 32 GB RAM and running Windows 10. The input for each training session consisted of a complex matrix of dimensions 880 × 880. The training process accounted for the physical pixel size of the spatial light modulator (SLM) by matching it to the spacing of the input matrix elements. Moreover, the model was trained with 200 epochs and was optimized using the built-in Adam optimizer. The learning rate was set to 0.1. Owing to the relatively small size of the network, training converged quickly (in 5 minutes on our used computer).

The resultant phase distribution of the diffraction layer was formatted as an 880 × 880 matrix, ranging from -π to π, aligning with the pixel resolution of the SLM, shown in Figure S2a. The evolution curve of the loss function with epoch is shown in Figure S2b.


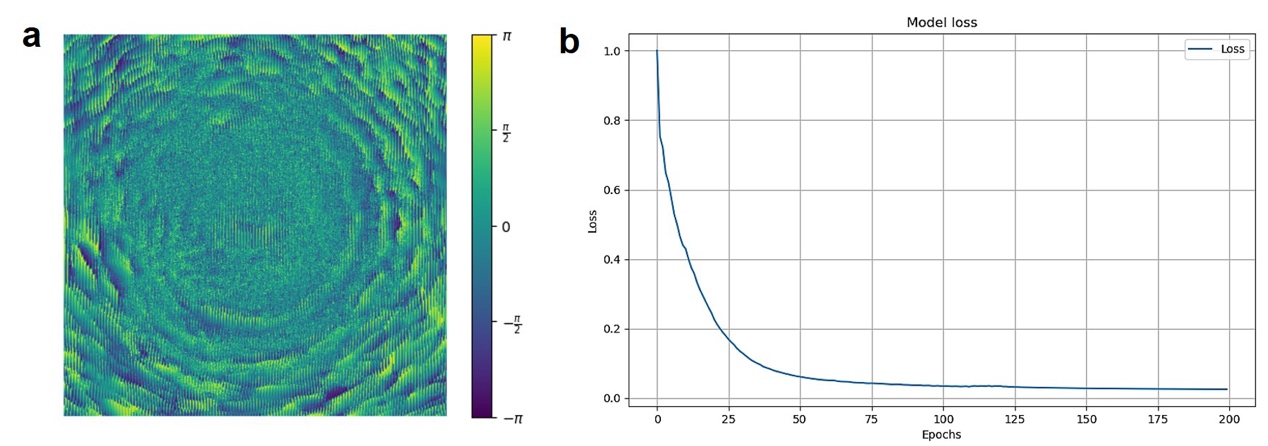


Figure S2. Training results. (a) The phase distribution obtained through training, and (b) The evolution of loss over epochs.

**Supplementary material 3: Coupling efficiency of the 3×1 beam combiner**

Table S1. The coupling efficiency of beam combiner.

| Channel | Power（W） | Efficiency（%） |
| --- | --- | --- |
| 1 | 27.1 | 97.5 |
| 2 | 27 | 97.1 |
| 3 | 27.1 | 97.5 |

A continuous laser was used to test the coupling efficiency of 3×1 beam combiner. The laser beam is coupled to each input port in turn, and probing with a power meter at the output. The input power is 27.8 W, output power and coupling efficiency of each port are shown in the Table S1.

**Supplementary material 4: The angular displacement curve of the micromirrors during the DMD switching process**

The technical manual of this DMD from the official website of Vialux (https://www.vialux.de/en/ultraspeed-v-modules.html) presents the angular displacement curve of the micromirrors during the DMD switching process, as shown in Figure S3. It can be observed that the entire switching process of the DMD lasts for 12 μs, consisting of 4 μs of reset time and 8 μs of settling time.


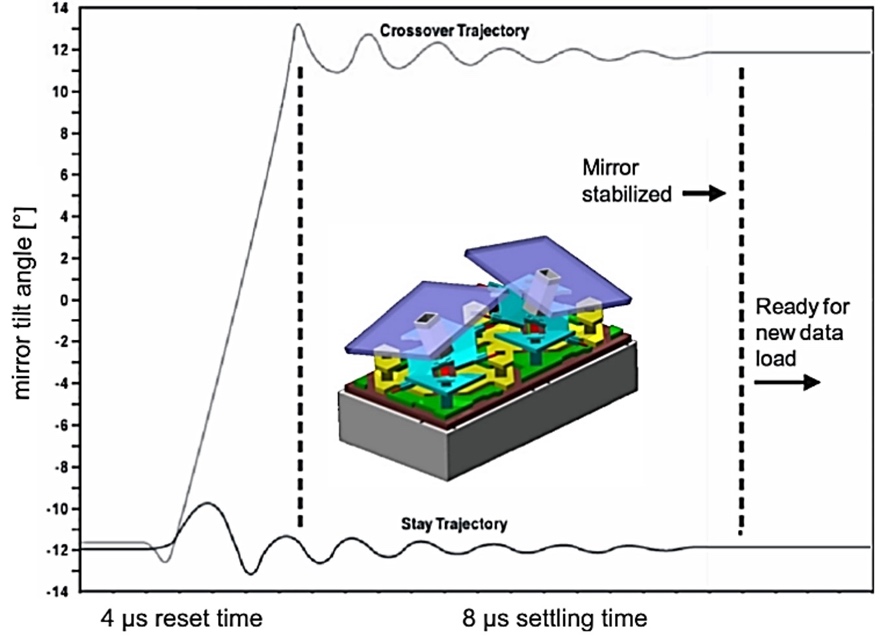


Figure S3. Global reset of the DMD.

**Supplementary material 5: Simulation results of multi-bit data in multi-layer networks**

To enable higher-bit demodulation, we conducted simulations using a multi-layer network, the hardware and parameter settings kept consistent with those used in the previous simulations (see Supplementary Material 2 for details). As shown in Figure S4a-d, we present the results of accurate 4-bit demodulation using a two-layer D^2^NN (only a subset of the results is shown due to their large volume). Figure S4e shows the two optimized diffractive layers.

Furthermore, we explored the effect of increasing the number of layers to assess the performance limit of the D^2^NN, as shown in Figure S4f. It is found that the demodulation capacity increases with the number of diffractive layers. However, the network reaches an upper limit of 7-bit demodulation when the number of layers exceeds five. This limitation arises because the D^2^NN inherently performs only linear operations without nonlinear activation functions. Further increasing the number of layers may lead to heightened system complexity and saturated computational capacity. To achieve higher performance, it is generally more effective to introduce nonlinear modules rather than simply stacking additional linear diffractive layers.


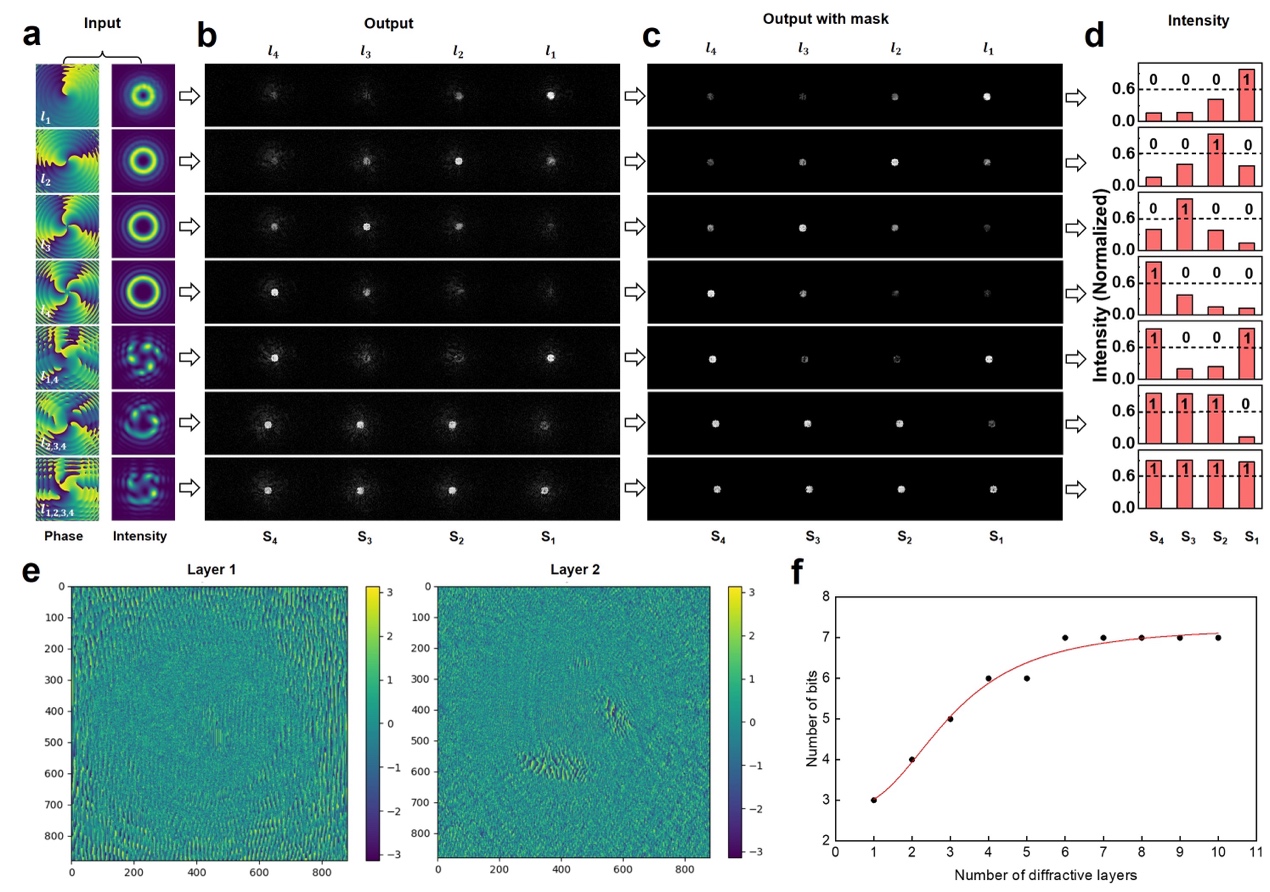


Figure S4. Simulated results of the multi-bit data demodulation. (a) Training dataset, including phase and amplitude from the complex matrix calculated by the binary holograms, (b) Output intensity distribution of the tow-layer D^2^NN, (c) Output intensity distribution with mask, whose sizes were designed to match four "activation regions", (d) Normalized intensity values through summarizing summing the light intensity in each "activation region", the intensity threshold was set at 0.6 to achieve 100% accuracy, (e) The phase distribution of the two trained layers, (f) The evolution of the number of bits over the number of diffractive layers.

**Supplementary material 6: Feasibility of replacing multimode fiber with single-mode fiber in our system (Note: Supplementary material 6 was prepared in response to the reviewers' comments and is not be cited in the main manuscript)**

In our proposed OAM-STM system, a pulsed laser source was employed. When the short pulses transmitted over extended distances through multimode fibers (MMFs), intermodal dispersion will become a significant concern. MMFs support multiple propagation modes, each traveling at slightly different group velocities. As a result, a single input pulse is effectively split into multiple components that arrive at the output at different times, leading to temporal broadening. As the pulse repetition frequency increases, the time interval between adjacent pulses of the output pulse sequences decreases. If the temporal broadening induced by intermodal dispersion exceeds the inter-pulse spacing, adjacent pulses begin to temporally overlap that severely affects accurate decoding of the transmitted information.

In our systems, each OAM mode corresponds to a time-encoded pulse, and an increase in the number of OAM channels effectively increases the density of the pulse train in the time domain. Consequently, the number of pulses per unit time increases, reducing the available guard interval between adjacent pulses. In this high-density regime, even moderate amounts of dispersion can result in substantial pulse overlap.

To mitigate this issue, one potential solution is to replace MMFs with single-mode fibers (SMFs) in the delay line components of the system. SMFs support only the fundamental mode, thereby eliminating intermodal dispersion. While chromatic dispersion still exists in SMFs, it is significantly weaker and more predictable.

Moreover, the use of SMFs offers additional advantages in terms of system stability and integration. For example, SMFs exhibit better mode confinement and lower susceptibility to environmental perturbations, such as bending or temperature fluctuations, which is especially beneficial in long-term or field-deployed systems. However, the trade-off includes stricter alignment requirements and potentially higher coupling losses, which must be balanced against the performance benefits.

In summary, although MMFs provide flexibility and simplified alignment for low-speed or short-distance systems, SMFs delay lines become essential in scenarios of high-repetition-rate pulse lasers and multiple OAM modes. This transition is critical to preserving temporal fidelity and ensuring the integrity of encoded data during transmission and demodulation.
